# Supplementary material for: Patterns of Intron Gain and Loss in Fungi
Source: PLoS Biol. 2004 Nov 30;2(12):e422. doi: 10.1371/journal.pbio.0020422 (PMC532390; doi:10.1371/journal.pbio.0020422)
Supplement: Table S1 — Also available at http://genes.mit.edu/NielsenEtAl/. (4.3 MB ZIP). [file pbio.0020422.st001.zip › NielsenEtAl/html/274.html]

AN1485.1.NCU03124.1.MG03696.1.FG00677.1


```
 CLUSTAL W (1.82) Multiple Sequence Alignments - Introns Inserted


Sequence 1: NCU03124.1	291 aa
Sequence 2: MG03696.1	338 aa
Sequence 3: FG00677.1	340 aa
Sequence 4: AN1485.1	333 aa
Alignment Length: 340 aa
Number Identitical Residues: 239 aa
Alignment Score (without introns) 10599


MG03696.1 	MARVYADVNANMPR~SYWEYDTVNIS~WGVLENYEVVRKIG1RGKYSEVFEGINVVNYQK
NCU03124.1	-----------MAR1-----------~--------------~RGKYSEVFEGINVVNYQK
FG00677.1 	MARVYADVNQSMPR~SYWDYDSVNIS1WGVLENYEVVRKIG1RGKYSEVFEGINVVNYQK
AN1485.1  	MARVYADVNKHMPR~SYWDYDSVNIS~WGVLENYEVVRKIG1RGKYSEVFEGINIVNYQK
          	 :   :. .  *.* :  . .: . :  .  .. .   . . ************:*****

MG03696.1 	CVIKVLKPVKKKKIKREIKILQNLAGGPNVVALLDVVRDSQ0SKTPSLIFEYVNNTDFRT
NCU03124.1	CVIKVLKPVKKKKIKREIKILQNLAGGPNIVALLDVVRDSQ~SKTPSLIFEYVNNTEFRT
FG00677.1 	CVVKVLKPVKKKKIKREIKILQNLAGGPNVVALLDVVRDSQ0SKTPSLIFEHVNNTDFRS
AN1485.1  	CVIKVLKPVKKKKIKREIKILQNLAGGPNVVALLDVVRDNQ~SKTPSLVFEYVNNTDFRT
          	**:**************************:*********.* ******:**:****:**:

MG03696.1 	LYPKFNDIDVRYYILELLKALDFCHSKGIMHRDVKPHN~VMIDHENRK~LRLIDWGLAEF
NCU03124.1	LYPRFNDFDVRYYIFELLKALDFCHSKGIMHRDVKPHN0---------~LRLIDWGLAEF
FG00677.1 	LYPKFNDIDVRFYIFELLKALDFCHSKGIMHRDVKPHN~VMIDHENRK0LRLIDWGLAEF
AN1485.1  	LYPRFSDYDVRFYIYELLKALDFCHSKGIMHRDVKPHN~VMIDHEKRK0LRLIDWGLAEF
          	***:*.* ***:** ***********************    . .. . ***********

MG03696.1 	YHPGTEYNVRVASRYFKGPELLVDYQEYDYSLDMWSLGAMFASMIFRKEPFFHGNSNSDQ
NCU03124.1	YHPGTEYNVRVASRYFKGPELLVDFQEYDYSLDMWSLGAMFASMIFRKEPFFHGQSNSDQ
FG00677.1 	YHPGTEYNVRVASRYFKGPELLVDFQEYDYSLDMWSLGAMFASMIFRKEPFFHGNSNSDQ
AN1485.1  	YHKGTEYNVRVASRYFKGPELLVDFQEYDYSLDMWSLGAMFASMIFRKEPFFHGNSNSDQ
          	** *********************:*****************************:*****

MG03696.1 	LVKIAKVLGTDDLFDYLDKYEIELDAQYDDILGRFQKKPWHSFVTAENQRFVSNEAIDFL
NCU03124.1	LVKIAKVLGTDELFDYLDKYEIELDAQYDDILGRFQRKPWHSFINAENQRFVSNEAIDFL
FG00677.1 	LVKIAKVLGTDDLFDYIDKYEIELDAQYDDILGRFQKKPWHSFVTSENQRFVSNEAIDFL
AN1485.1  	LVKIAKVLGTEELFEYLDKYEIELDPQYDEILSRFPRKPWQSFVNAENQRFISDEAIDFL
          	**********::**:*:********.***:**.** :***:**:.:*****:*:******

MG03696.1 	DKLLRYDHM0ERLTAKEAMAHPYFEPVRGEGVLERLLAAGTNTSA--
NCU03124.1	DKLLRYDHN0ERLTAKEAMAHPYFAPVRDEATRARYLAGETIN----
FG00677.1 	DKLLRYDHQ0ERLTAKEAQAHPYFNPVRDPEVFKQHLAAASGAANKS
AN1485.1  	DKLLRYDHA0ERLTAQEAMAHPYFAQIRAEEAANRSTASS-------
          	********  *****:** *****  :*   .  :  *.
```
